# Exergy Analysis of Coal-Based Series Polygeneration Systems for Methanol and Electricity Co-Production

**DOI:** 10.3390/molecules26216673

**Published:** 2021-11-04

**Authors:** Jianyun Zhang, Zhiwei Yang, Linwei Ma, Weidou Ni

**Affiliations:** 1 China Energy Technology and Economics Research Institute, China Energy Investment Corporation Ltd., Beijing 102211, China; jianyun.zhang.c@chnenergy.com.cn; 2 Department of Energy, Environmental & Chemical Engineering, Consortium for Clean Coal Utilization, Washington University in St. Louis, One Brookings Drive, St. Louis, MO 63130, USA; 3 Tsinghua-BP Clean Energy Center, State Key Laboratory of Power Systems, Department of Thermal Engineering, Tsinghua University, Beijing 100084, China; malinwei@tsinghua.edu.cn (L.M.); niwd@tsinghua.edu.cn (W.N.)

**Keywords:** coal, polygeneration, methanol, power, exergy, methanol, potential efficiency

## Abstract

This paper quantifies the exergy losses of coal-based series polygeneration systems and evaluates the potential efficiency improvements that can be realized by applying advanced technologies for gasification, methanol synthesis, and combined cycle power generation. Exergy analysis identified exergy losses and their associated causes from chemical and physical processes. A new indicator was defined to evaluate the potential gain from minimizing exergy losses caused by physical processes—the degree of perfection of the system’s thermodynamic performance. The influences of a variety of advanced technical solutions on exergy improvement were analyzed and compared. It was found that the overall exergy loss of a series polygeneration system can be reduced significantly, from 57.4% to 48.9%, by applying all the advanced technologies selected. For gasification, four advanced technologies were evaluated, and the largest reduction in exergy loss (about 2.5 percentage points) was contributed by hot gas cleaning, followed by ion transport membrane technology (1.5 percentage points), slurry pre-heating (0.91 percentage points), and syngas heat recovery (0.6 percentage points). For methanol synthesis, partial shift technology reduced the overall exergy loss by about 1.4 percentage points. For power generation, using a G-class gas turbine decreased the overall exergy loss by about 1.6 percentage points.

## 1. Introduction

Coal-based polygeneration, which integrates chemical production and power generation, is a promising strategy for clean coal utilization [1,2]. Today’s coal-based polygeneration systems can potentially be significantly improved by systematic optimization [3,4] and by applying advanced technical approaches [5,6,7]. For example, in a methanol-power polygeneration system, the system comprises coal gasification, methanol synthesis, and combined cycle power generation. Applying advanced technical refinements to each of these components can potentially improve the energy efficiency of the entire system, as summarized below.

For gasification, recovering the sensible heat of syngas from the gasifier can reduce energy loss. Hot gas cleaning technology represents another attractive solution to improve energy efficiency [8,9,10], and an approximately 2.5 percentage point efficiency improvement has been reported [8]. Coal-water-slurry pre-heating technology offers another opportunity to boost energy efficiency during gasification [11], and ion transport membrane technology for air separation can significantly reduce the energy consumption for oxygen production [12,13,14].

For methanol synthesis, different process configurations can lead to different plant efficiency values. For example, the system performance can be strongly influenced by: (1) recycling the unreacted syngas back to the methanol reactor; and (2) adjusting the syngas composition downstream of gasification using a water gas shift reactor [2]. For combined cycle power generation, applying an advanced G-Class gas turbine can improve power generation efficiency.

Although the benefits of the above technologies and process optimizations have been explored individually in different studies [2,8,9,10,11,12,13,14], it is worth noting that these studies were performed based on different polygeneration systems. A systematic analysis is needed to compare the impacts of all these technical approaches on a common design basis so that the relative importance of these approaches and the maximum potential efficiency of a polygeneration system can be appropriately evaluated. The goal of this work is to address this need.

For a polygeneration system that co-produces electricity and liquid fuel, it is difficult to assess the system performance using a conventional evaluation indicator like energy efficiency. Alternative evaluation methods have been developed in the literature, among which exergy analysis is one of the most popular. There are three categories of exergy analysis in the literature:

(1) Simple exergy analysis. This method quantifies system performance by accounting for the overall irreversibility of the entire system, as described by Gangadharan [15] and Bhattacharya [16]. It evaluates exergy balance on the macro level and ignores the detailed information of exergy destruction (i.e., location, amount, and mechanism).
(2) Structured exergy analysis. This method assesses the exergy balance on a component level (such as gasifier, gas turbine), such as in Kunze [17], Wang [18], and Gao [19]. Thus, it can help process developers identify the locations and amounts of exergy losses in each component. However, for a complicated system like polygeneration, the process in which the largest exergy loss occurs may not be the one where the easiest reduction of exergy loss can be achieved. For example, the gasifier has a significant exergy loss due to chemical reactions, but this exergy loss is inevitable in a polygeneration system.
(3) Physical-chemical process exergy analysis. This method analyzes the exergy balance based on each physical or chemical subprocess, such as in Liu [20]. This method moves one further step compared with the structured exergy analysis. It not only identifies where exergy destruction occurs but also why it occurs. By knowing the mechanisms of the exergy losses, we can evaluate the feasibility and level of difficulties in reducing these exergy losses.

This study will use the physical-chemical process exergy analysis to evaluate the locations and mechanisms of exergy losses in a coal-based polygeneration system that co-produces methanol and electricity. In addition, by analyzing a series of advanced component technologies, we explored the relative contribution of each technology and the maximum potential efficiency of the polygeneration system. Section 2 presents the exergy analysis method and an indicator we defined to evaluate the exergy utilization efficiency of physical processes. Section 3 introduces the model used for process simulation: Aspen Plus is used to model the chemical production process, and GT Pro is used to model the power generation process. In Section 4, the distribution of exergy loss in the system is mapped, and the impacts of different advanced component technologies are presented. Finally, the potential reduction of overall exergy loss by applying these technical solutions is quantified. Section 5 discusses simulation results and provides conclusions.

## 2. Methodology

### 2.1. Polygeneration System Description

**Figure 1:**
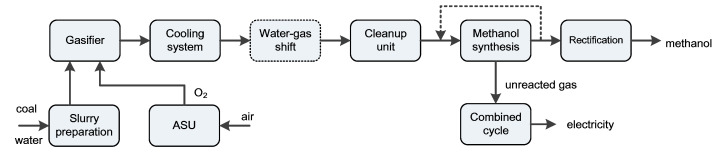
A series polygeneration system based on coal gasification.

**Figure 2:**
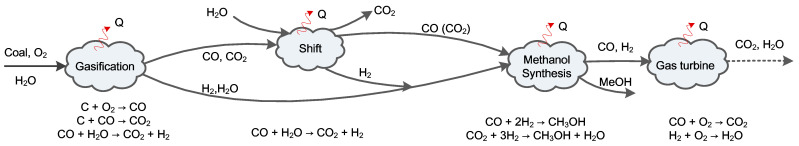
Energy and material conversion in a coal-based polygeneration system.

Figure 1 illustrates the basic configuration of a typical coal-gasification-based series polygeneration system co-producing methanol and electricity. This polygeneration system is composed of a chemical production block (including units for slurry preparation, gasification, air separation, cooling, water-gas shift, acid gas removal/cleanup, methanol synthesis, and rectification) and a power generation block (including a gas turbine, heat recovery steam generator, and steam turbine). The outputs are controllable, as indicated by the dotted line. The material conversion and energy transformation in the system are illustrated in Figure 2. There are four main processes where substance conversion occurs simultaneously with heat transfer: gasification, water-gas shift, methanol synthesis, and combustion inside the gas turbine. Exergy is partially destroyed in each of these processes, and other heat transfer and separation processes also cause exergy destruction.

This paper will quantitatively analyze the total potential efficiency improvement for the above coal-based polygeneration system through: (1) adopting advanced technologies for the gasification block, (2) optimizing the process design for the methanol synthesis, and (3) selecting an advanced combined cycle. The polygeneration cases analyzed in this paper are listed as follows:

- Base case: a series polygeneration system with water-gas shift and a once-through methanol reactor, as shown in Figure 1.
- Cases with improved technical solutions for gasification: (1) sensible heat recovery unit, (2) hot gas cleaning technology, (3) coal water slurry pre-heating vaporization technology, and (4) ion transport membrane for air separation.
- Cases with advanced process designs for methanol synthesis: (1) partial shift, i.e., the H_2_/CO ratio fed to methanol reactor is lower than the stoichiometric ratio; and (2) unreacted gas circulation.
- Cases with advanced gas turbines for the combined cycle: (1) F-class gas turbine, and (2) advanced G-class gas turbine.

### 2.2. Physical-Chemical Process Exergy Analysis

In the method followed in this work, each critical process is divided into a series of simple physical or chemical sub-processes, then an exergy balance is built for each sub-process, and the associated exergy loss is calculated. This process decomposition reveals the internal mechanisms of exergy losses.

Our example polygeneration system, as discussed above, is composed of several complex processes that can be classified into six categories: (1) chemical reactions with drastic changes of temperature; (2) gas-to-steam heat transfer, such as radiant syngas cooling and convective syngas cooling; (3) gas-gas or gas-liquid mixing with heat and mass transfer, such as quenching and scrubbing processes; (4) low-temperature cooling with water liquefaction; (5) species separation, such as oxygen generation from air separation and acid gas removal; and (6) chemical reactions occurring at a constant temperature, such as methanol synthesis and water gas shift reactions. Each of these categories is described in detail below.

**Figure 3:**
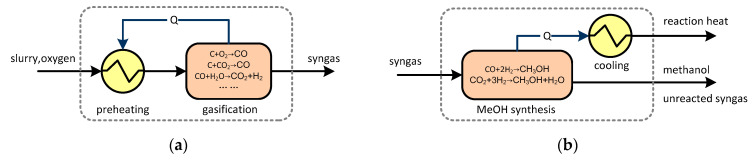
Exergy balance of several key components in polygeneration systems. (**a**) Gasifier; (**b**) Methanol synthesis.

(1) As examples of chemical reactions with drastic temperature changes, the gasification reactions in the gasifier and the combustion reactions in the combustor of the gas turbine belong to the first category. Take the gasification process as an example. As shown in Figure 3a, the gasification process can be hypothetically decomposed into two sequential sub-processes: first, the reactants are heated by hot products to the reaction temperature *T*; second, the fuel reacts with pure oxygen at a constant temperature. The exergy balance is given by Equation (1), where *I_chem_*, *I_heating_*, and *I_Qloss_* are exergy destructions caused by the gasification reactions, reactant heating, and heat loss in the gasifier, respectively:

(1) $$E_{coal,in}+E_{O_{2},in}=E_{syngas,out}+E_{slag,out}+I_{chem}+I_{heating}+I_{Qloss}$$

(2) $$I_{chem}=\left(E_{out}-E_{in}\right)-\Delta H\cdot \left(1-T_{0}/T\right)$$

(3) $$I_{heating}=T_{0}\cdot \Delta s_{heating}=T_{0}\cdot \left(s_{out}-s_{in}-\left(h_{out}-h_{in}\right)/T\right)$$

(4) $$I_{Qloss}=E^{Q}=Q_{loss}\cdot \left(1-T_{0}/T\right)\;$$

(2) The heat transfer from hot syngas to steam leads to exergy loss, *I_heating_*, and the exergy balance is presented in Equation (5), where *I_Qloss_* represents the exergy loss due to heat dissipation to the atmosphere.

(5) $$E_{water}+E_{syngas,in}=E_{steam}+E_{syngas,out}+I_{heating}+I_{Qloss}$$

(3) In the third category, water is vaporized and mixed with syngas. Taking the quenching process as an example, the exergy balance is expressed in Equation (6):

(6) $$E_{water,in}+E_{syngas,in}=E_{water,out}+E_{syngas,out}+I_{Qloss}$$

(4) In low-temperature cooling, the exergy balance can be given by Equation (7), and the thermal energy is divided into sensible and latent heat. When the temperature decreases, part of the steam in the syngas condenses, releasing a large amount of latent heat, which is quantified by Equation (8).

(7) $$E_{syngas,in}=E_{syngas,out}+E_{water}+E_{latent}^{Q}+E_{sensible}^{Q}$$

(8) $$E_{latent}^{Q}=\int_{x_{in}}^{x_{out}}\Delta H\cdot \left(1-T_{0}/T\right)\cdot dm_{water}$$

(9) $$E_{sensible}^{Q}=E^{Q}=Q\cdot \left(1-T_{0}/T\right)$$

(5) The exergy balance of a separation process can be described by Equation (10), and the theoretical minimal separation work, $E_{AGR}^{w}$, is estimated by Equation (11). The exergy efficiency is set to be 20%, so the actual heat exergy consumed in the separation process is about five times the theoretical minimal separation work.

(10) $$E_{syngas,in}+E_{AGR}^{w}/\eta =E_{syngas,out}+E_{acidgas,out}+I_{loss}$$

(11) $$E_{AGR}^{w}=RT_{0}\cdot \left(n_{CO_{2}}\cdot ln\left(\frac{n_{CO_{2}}}{n_{CO_{2}}+n_{other}}\right)+n_{other}\cdot ln\left(\frac{n_{other}}{n_{CO_{2}}+n_{other}}\right)\right)$$

(6) The methanol synthesis reaction and water gas shift reaction can be classified in the sixth category, reacting at a constant temperature. Equation (12) describes the exergy balance of these reactions, where E^Q^ represents the heat exergy discharged from the reactor and *I_chem_* represents the exergy expended to drive the chemical reactions.

(12) $$E_{syngas,in}=E_{syngas,out}+I_{chem}+E^{Q}$$

### 2.3. Physical Exergy Efficiency

In previous studies, the energy utilization of polygeneration systems has often been quantified by exergy efficiency, which is defined as the ratio of the total output exergy to the total input exergy and expressed by Equation (13), where *E_p_* is the exergy of the electricity and *E_c_* is the exergy of the methanol product:

(13) $$\eta =\left(E_{P}+E_{c}\right)/E_{total}.$$

The electricity exergy is equal to its thermal equivalent, while the chemical exergy of the methanol is equal to its heating value. From the exergy perspective, power and chemical fuels can be considered the same. In other words, the exergy efficiency of a polygeneration system is equivalent to its energy efficiency. Furthermore, because the thermal efficiency of methanol production is about 10 percentage points higher than that of an IGCC (integrated gasification combined cycle) plant, the overall exergy efficiency depends on the product ratio. Thus, it is inappropriate to use exergy efficiency to compare different polygeneration systems with different product ratios. 

In a polygeneration system, several reactions produce chemicals instead of providing thermal energy, such as gasification, water gas shift, and methanol synthesis reactions. The exergy losses in driving these chemical reactions are expenses for the desired substances. Hence, these exergy losses are inevitable. On the contrary, the exergy losses in physical processes like heat transfer can potentially be reduced by properly designing the heat network or sacrificing the economics of the system. Therefore, from the perspective of energy utilization, more attention should be focused on minimizing the physical exergy losses.

This paper presents a new indicator to evaluate the physical exergy utilization in polygeneration systems, defined by Equation (14):

(14) $$\varphi =E_{P}/\left(E_{total}-E_{c}-I_{chem}\right),$$

where *I_chem_* is the exergy loss for driving the chemical reactions in a polygeneration system, which can be quantified based on a structured exergy analysis. The denominator represents the total amount of exergy which might be consumed by physical processes, which is calculated by subtracting the exergy changes in substance conversion processes from the total exergy input. The physical exergy efficiency, *φ*, indicates the level at which the physical exergy is utilized in a whole polygeneration system, and it is suitable for assessing polygeneration systems with different product ratios.

## 3. System Modeling

This paper integrates the commercial software Aspen Plus and GT Pro for process simulation. The former is used to model the chemical production block, while the latter is used to model the power generation block.

The property method used in Aspen Plus is Peng-Robinson with the Boston-Mathias alpha function (PR-BM). Coal is considered a nonconventional solid substance in Aspen, and it is assumed to be decomposed into available components, including C, H_2_, O_2_, etc., before participating in gasification reactions. The reactions are assumed to reach equilibrium, and the syngas composition can be calculated based on the Gibbs free energy minimum principle. The hot products are then quenched directly by cold water, where some physical exergy loss occurs. Fine particles and ammonia in the syngas are removed in the scrubber.

In the water gas shift reactor, the ratio of H_2_ to CO in the syngas is adjusted to 2.10–2.15 via CO + H_2_O → H_2_ + CO_2_ for the subsequent methanol synthesis. This reaction is exothermal, leading to a 100–200 °C temperature increase in the reactor. Therefore, in the exergy analysis below, the water gas shift process is considered an isothermal process for simplification.

The acid gas removal unit is operated approximately at ambient temperature. This unit comprises an absorption tower and a regeneration tower modeled by the RadFrac block in Aspen. H_2_S is completely removed, and the CO_2_ concentration of the sweet gas is reduced to 3% to benefit methanol synthesis.

The Aspen Plus RCSTR block, assuming perfect mixing in the reactor, is used to simulate the methanol synthesis process. Here the Langmuir–Hinshelwood–Hougen–Watson (LHHW) model is selected to specify the reaction parameters, and the kinetic data for the catalyst, C301, is used [21]. In this reaction, the thermal energy is released and removed to maintain a constant operating temperature. The cold reactant gas is pre-heated by the hot products. The produced raw methanol is subsequently purified in a triple-tower distillation unit driven by intermediate-pressure steam.

The combined cycle is modeled in GT Pro, a tool for simulating power generation processes. The selected gas turbine is GE 9531FA model, and the inlet guide vanes (IGV) are adjusted by about 8% so that the mass flow rate of the compressor matches that of the turbine, offsetting the mass flow increase due to fuel injection. The steam cycle includes a three-pressure reheat heat recovery steam generator (HRSG) and steam turbines. As discussed later, the additional steam generated in the gasification block is integrated with the main stream in improved cases. The operating parameters and model specifications are summarized in Table 1.

## 4. Results and Discussion

### 4.1. Model Validation

In this section, models for three key components of the coal-based poly-generation system-gasification, methanol synthesis, and gas turbine—are validated against literature data. 

(1) Validation for the gasifier model. The gasifier model was used to predict the syngas compositions of different entrained flow gasifiers operating with different coals. The operating conditions and coal properties were obtained from the literature [22,23,24]. Table 2 shows a comparison between industrial data and model results. As shown, the main syngas compositions predicted by the model agree reasonably well with the industrial data. Accurately predicting the gasifier performance requires a comprehensive kinetic model that fully accounts for the dependence of reactor geometry, burner configuration, coal type, etc. This work focuses on plant-level analysis and thus used a simple thermodynamic model with an assumed carbon conversion ratio for simplicity. For industrial-scale entrained flow gasifiers, the syngas exiting the gasifer is generally at or near local chemical equilibrium. Therefore, a simple thermodynamic gasifier model can usually provide sufficient accuracy for process analysis. In Table 2, the CO_2_ level of the Wu [24] case has a relatively large deviation because the gasifier carbon conversion ratio in this case is relatively low. 

(2) Validation for the methanol synthesis model. The experimental results of a slurry-based methanol synthesis pilot unit at Laporte [25] were used to validate the methanol synthesis model in this work. Ten operating conditions were simulated, as shown in Table 3. 

The methanol equivalent productivities predicted by the model agree reasonably well with the experiment results, except for conditions No. 4 and No. 6, which operate at extremely low space velocities. Low space velocity leads to low heat transfer rate and low methanol productivity and thus is generally avoided in industrial designs. For simplicity, our model focused on normal space speed operation and neglected the effect of space velocity on heat transfer for simplicity.

(3) Validation of the gas turbine model. The gas turbine model used in this work has been developed and compared with data generated by GT PRO in our previous study [26]. GT Pro is design software that has been widely used in the gas turbine industry. The accuracy of GT Pro is well accepted by the industry. Our previous work [26] showed that the performance of an air-cooled gas turbine predicted by our model, including the net power output, efficiency, and exhaust temperature, is consistent with that from GT Pro.

### 4.2. Results of the Base Case

Mass and energy balance calculations were performed based on the above model, followed by an exergy analysis. The results are presented in the following sequence: (1) base case, (2) effect of technical solutions for the gasification block, (3) effect of designs for the methanol synthesis, (4) effect of gas turbine choices, and (5) the calculated potential efficiency improvement in the polygeneration system.

**Figure 4:**
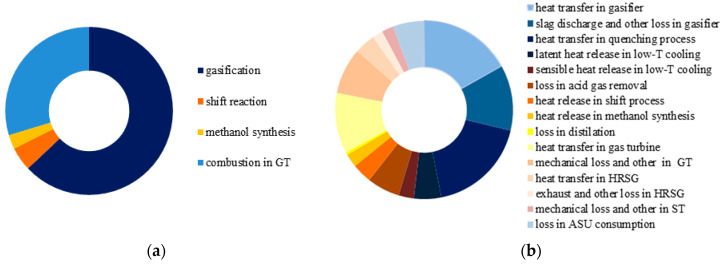
Structure of exergy loss in the base case. (**a**) chemical processes; (**b**) physical processes.

For the base case, the irreversible exergy losses caused by chemical reactions account for about 21% of the total exergy input, while the irreversible losses caused by physical processes account for 36%. Figure 4a indicates that about 70% of the exergy destruction caused by chemical processes comes from the gasification reactions, followed by the combustion reactions in the gas turbine, water gas shift reactions, and methanol synthesis reactions. For physical processes, as shown in Figure 4b, the primary exergy destruction is caused by the heat transfer and slag discharge in the gasifier, heat release in the quenching process, and heat transfer in the combustor of the gas turbine.

### 4.3. Technical Solutions for Gasification

#### 4.3.1. Sensible Heat Utilization

In the base case, the hot syngas from the gasifier is directly quenched by cold water, leading to a large exergy loss (6.574% of the total exergy input). However, the sensible heat of the syngas above 800 °C can be recovered for steam generation by a radiant syngas cooler (RSC) [27]. The intermediate-temperature syngas after the RSC can be quenched by water to increase the syngas moisture content and facilitate downstream water-gas shift processes, where water is a reactant and eliminates the need for an expensive convective cooler.

When there is an RSC, the exergy loss in the quenching process decreases to 3.387% of the total exergy input. The loss in the RSC is about 1.759%, as shown in Table 4. Thus, heat recovery can effectively reduce exergy loss. Also, the heat release in the low-temperature cooling process declines from 3.361% to 1.773% due to lower steam content in the quenched syngas. Nevertheless, the exergy loss caused by heat transfer in the HRSG is doubled due to the increased steam amount from quenching upstream of the HRSG. The net effect of sensible heat utilization is a 1.472 percentage point reduction of the overall exergy loss.

#### 4.3.2. Hot Gas Cleaning Technology

The raw syngas from the gasifier contains many solid particles and acid gases that could be removed at a higher temperature to achieve optimal energy efficiency and protect downstream devices. Therefore, hot gas cleaning (HGC) is a critical process that can avoid the energy-intensive cooling and heating processes occurred in a conventional gas cleaning (CGC) unit at atmosphere temperature [10,28]. When an HGC is employed, the physical processes downstream of the gasifier are influenced. In the HGC case, the raw syngas is first cooled down. Then the halides and alkalis in the flue gas are separated by adding sorbents, and sulfur components are removed by a zinc titanate sorbent at 650 °C. Most fine particles are finally captured by the filter [8,29,30]. It should be noted that, for a fair comparison, in the CGC case, the raw syngas is also first cooled down to 800 °C via RSC.

**Figure 5:**
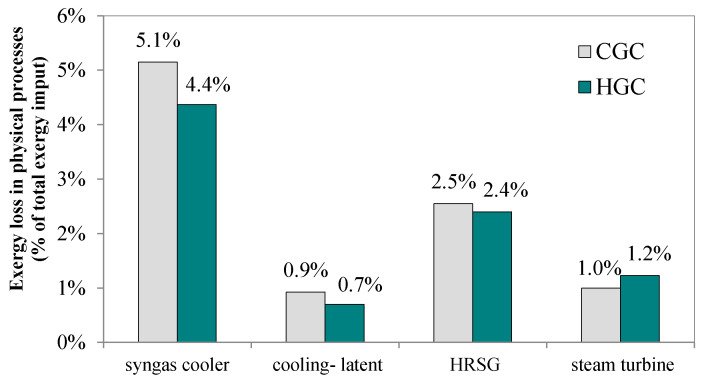
Exergy loss of physical processes when employing HGC.

Figure 5 shows HGC technology’s impact on the exergy losses of the polygeneration system. 14.4% less exergy is lost during syngas cooling in the HGC case, reduced from 5.1 percentage points to 4.4 percentage points because the high-temperature desulfurization and solid removal recover the thermal energy of the hot syngas in the intermediate temperature range. 

This thermal energy would otherwise be lost to the cold water in the CGC case. Furthermore, because the water content in the quenched syngas is less than that in the quenching process, less latent heat is lost. Thus, in general, employing the HGC technology can reduce the overall exergy loss of a polygeneration system by approximately 4.3%.

#### 4.3.3. Coal Water-Slurry Pre-Heating Vaporization Technology

**Figure 6:**
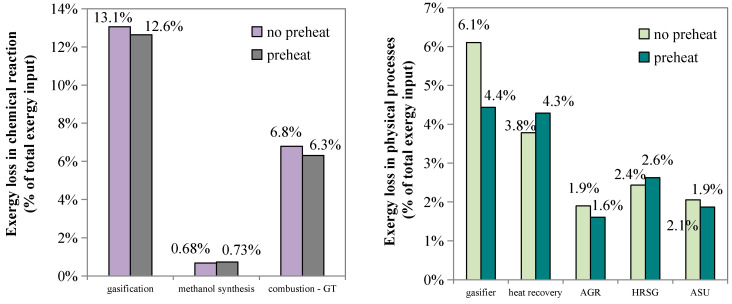
Exergy losses in chemical reactions and physical processes with and without slurry pre-heating.

Pre-heating coal water-slurry provides an opportunity to enhance the efficiency of wet-fed gasifiers, according to Zhang [31]. The main influence of this technology on exergy losses caused by chemical and physical processes is illustrated in Figure 6. Without slurry pre-heating, the exergy loss in chemical reactions is 13.1 percentage points in gasification, 0.68 percentage point in methanol synthesis, and 6.8 percentage points in the combustion reactions occurring in gas turbine, while with slurry pre-heating, the above exergy losses are decreased to 12.6 percentage points, 0.73 percentage point, and 6.3 percentage points, respectively. The exergy loss caused by chemical reactions decreases from 20.5 percentage points to 19.7 percentage points, while that caused by overall physical processes decreases to 33.6 percentage points from 34.2 percentage points.

In the slurry pre-heating case, the exergy loss caused by gasification reactions is reduced by 3%, while that caused by reactant pre-heating in the gasifier is reduced by 27% compared with the no-preheat case. Because the pure oxygen demand of gasification is decreased, 12% less exergy is lost in the ASU. However, more exergy is lost in the sensible heat utilization process because the portion of the sensible heat of the syngas used for steam generation in the no-preheat case is used here for vaporizing the low-temperature slurry. Due to slurry pre-heating, the desirable CO and H_2_ components increase, and H_2_O and CO_2_ decrease. Thus, the exergy losses that occur downstream vary accordingly. For instance, the exergy loss of acid gas removal falls by about 15%, but the exergy loss caused by combustion reactions in the gas turbine increases by about 7%. Overall, slurry pre-heating can decrease the exergy loss of a polygeneration system by approximately 2.7%.

#### 4.3.4. Ion Transport Membrane

In the previous simulation, cryogenic air separation was adopted since this technology has already been commercialized and is widely employed in the chemical industry. The specific energy consumption of a large-scale ASU is reported to be between 0.22 and 0.27 kWh/kg O_2_ [32]. In this paper, it is set to be 0.25 kWh/kg O_2_. The exergy loss in this separation process accounts for about 2.05% of the total exergy input and mainly occurs in the feedstock pre-processing and distillation section [33]. The ion transport membrane (ITM) is a promising technology for oxygen production, with better economics and efficiency than an ASU. The theoretical specific energy consumption of the air separation process is calculated to be 0.052 kWh/kg O_2_ in this paper, close to the 0.049 kWh/kg O_2_ provided by Fu [34]. According to Mancini [13], the total work loss in an ITM process varies between 0.034 and 0.064 kWh/kg O_2_. When this work loss is combined with the theoretical specific energy consumption, the total exergy consumption should be between 0.086 and 0.116 kWh/kg O_2_. In this work, 0.11 kWh/kg O_2_ was chosen as the specific exergy consumption for the ITM. Ultimately, about 70% less exergy is lost in the ITM process than conventional cryogenic air separation.

### 4.4. Technical Choices in the Methanol Synthesis Process

This section analyzes the influence of different methanol synthesis technical choices on the overall exergy loss. Two aspects are involved: partial water-gas shift and unreacted syngas circulation.

#### 4.4.1. Partial Water Gas Shift Process

**Figure 7:**
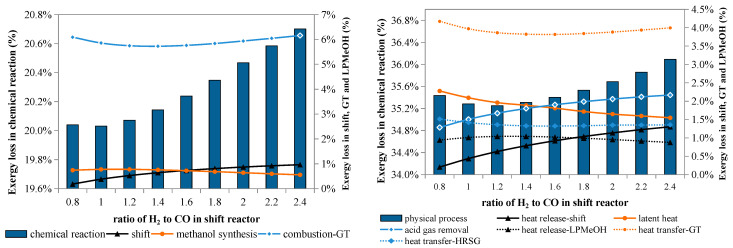
Exergy loss in a partial shift polygeneration system.

Figure 7 illustrates the effects of the water gas shift process on exergy losses in chemical reactions and physical processes. Both exergy losses declined compared with the base case, where the H_2_/CO ratio after the water gas shift process is approximately equal to the stoichiometric value (about 2.4).

When the amount of the shifted gas decreases, the exergy loss caused by the shift reaction goes down accordingly; meanwhile, the exergy loss caused by the methanol reactions first rises, then decreases, while that caused by the combustion reactions first falls, then goes up. Both variations occur in a narrow range because the conversion of CO and H_2_ in the Liquid-Phase Methanol Process (LPMeOH) reactor is dependent on the composition of the reactant gas.

A lower H_2_/CO ratio means that less syngas is shifted, and consequently less CO_2_ is generated and needs to be separated before the methanol synthesis; as a result, less exergy is lost in this separation process, which is otherwise a major contributor to the exergy lost to physical processes. Additionally, the exergy loss caused by heat release in the shift reactor also declines. These two causes can completely offset the increased exergy loss in other processes.

**Figure 8:**
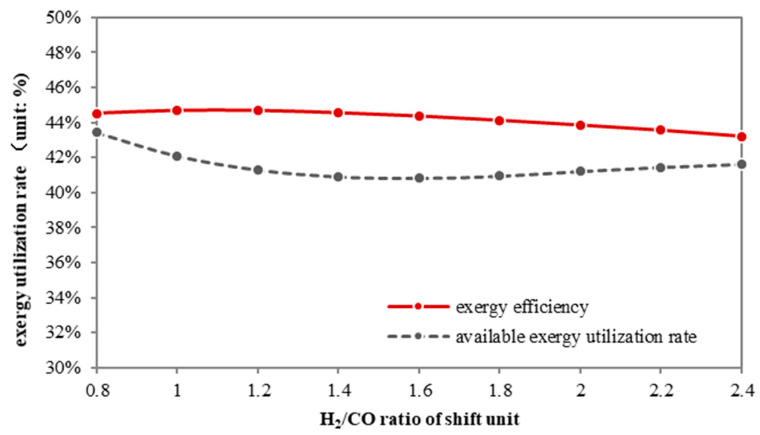
Exergy efficiency and physical exergy efficiency of polygeneration w/partial shift.

As shown in Figure 8, the partial shift process benefits both overall and physical exergy efficiency. The overall exergy efficiency of a partial shift system grows from 43.5% to 44.6% when the H_2_/CO ratio decreases, while the physical exergy efficiency first falls, then rises. Although these two curves are not completely consistent, the trend indicates that the less the syngas is shifted, the more efficiently the thermal exergy is utilized.

When the water gas shift reactor is completely omitted, syngas without any composition adjustment is directly fed to the methanol production process. In other words, the raw material for the methanol reaction is rich in CO, which can be regarded as an extreme case of the partial shift case with no shifted gas. The results are shown in Table 5. 

The exergy losses caused by the exothermal shift process (0.95 percentage point) are avoided, including those for driving the chemical reaction and the heat released from this reaction (1.29 percentage points). Because of the inappropriate reactant composition (i.e., rich in CO), about 21% more exergy is wasted in the methanol synthesis, rising up from 0.56 percentage point to 0.68 percentage point, leading to lower CO conversion and lower methanol production. Because more unreacted gas is discharged from the methanol reactor, exergy losses in downstream physical processes increase only modestly, as shown in the last five items in Table 5. Besides, since the CO_2_ concentration at the inlet of the acid gas removal unit is 60% lower than that of the base case, the exergy loss in the separation process is consequently reduced from 2.16 percentage points to 1.06 percentage points. In brief, omitting the shifting process benefits the exergy utilization of the whole system, and the total exergy loss decreases by 2.4 percentage points.

#### 4.4.2. Unreacted Gas Circulation

**Figure 9:**
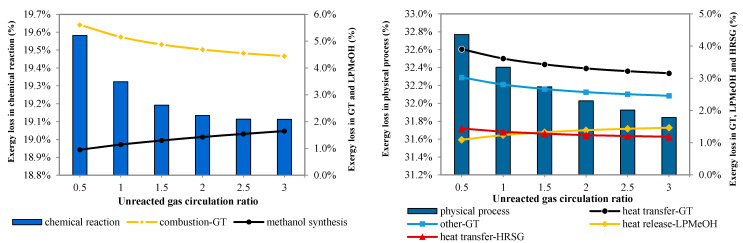
Exergy loss of chemical processes in the no-shift series polygeneration system.

Unreacted gas circulation does not affect the upstream processes, so here we focus only on the methanol synthesis process and combined cycle. Figure 9 shows the effects of the unreacted gas circulation ratio (λ) on exergy losses caused by chemical reactions and physical processes. λ is defined as the ratio of the mass flow rate of the recycled gas to that of the fresh gas entering the methanol reactor. The exergy loss decreases with λ, that is, enhancing the unreacted gas recycle benefits the exergy efficiency.

With higher λ, the exergy loss caused by the combustion reactions is lower because the amount of fuel gas is reduced; meanwhile, the exergy loss caused by the methanol synthesis reaction increases slightly due to the increased syngas in the reactor. However, the net effect is that the total exergy loss in chemical reactions decreases. For the physical processes, because of less fuel gas, the exergy losses caused by heat transfer in the gas turbine and HRSG are reduced when λ rises; for the same reason, the exergy loss from heat release in the LPMeOH reactor also declines.

**Figure 10:**
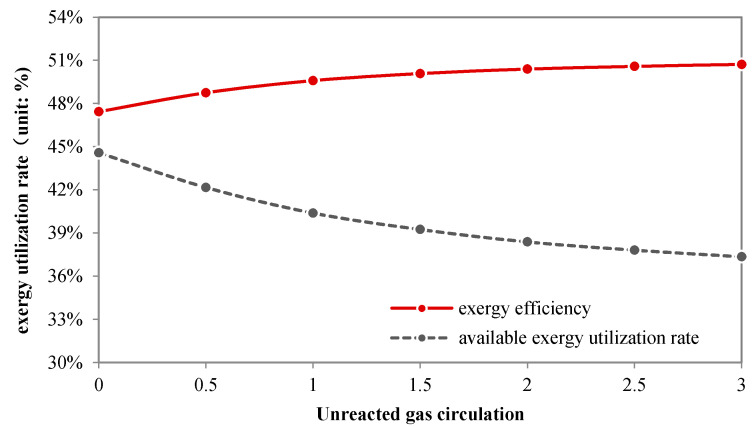
The exergy utilization rate of the no-shift series polygeneration system.

Figure 10 illustrates the effect of λ on the overall exergy efficiency and physical exergy efficiency. 

The exergy efficiency increases with λ, which can be partly attributed to the increased methanol production. On the contrary, the physical exergy efficiency falls by 5%. Thus the thermal energy utilization becomes worse. Therefore, from the perspective of physical exergy utilization, unreacted syngas circulation is not an advisable choice.

### 4.5. Technical Choices for the Gas Turbine

The gas turbine is a key component of the power generation block, using the upstream syngas to produce electricity. Two different gas turbines are selected here, an F-class design and an advanced G-class one. The latter has a higher turbine inlet temperature of 1500 °C and also a higher pressure ratio of 20. In this section, we analyze and compare the influence of different gas turbines on the energy utilization of a polygeneration system.

**Figure 11:**
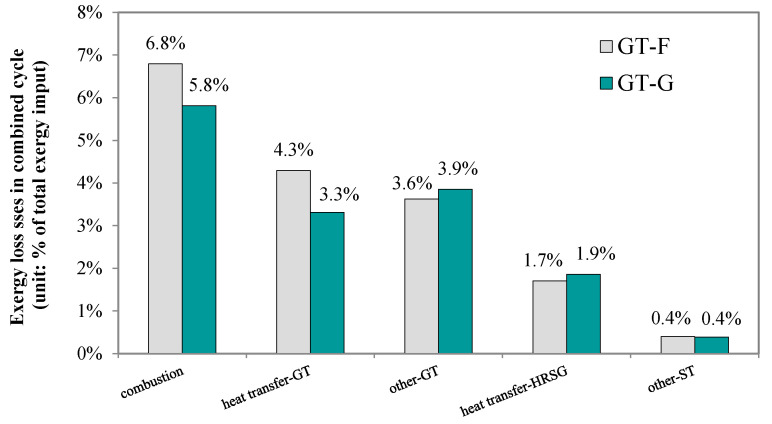
Effect of gas turbine choices on exergy losses of polygeneration systems.

As Figure 11 indicates, with the G-class gas turbine, the exergy loss caused by the air heating process in the combustor is reduced from 4.3 percentage points to 3.3 percentage points, due to the lower inlet mass flow of the compressed air. The exergy loss caused by the combustion reactions declines by 15%, while the other losses, including the mechanical loss in the gas turbine and heat transfer loss in the HRSG, increase by about 10%. The net effect is that the overall exergy loss of the system with a G-class turbine is reduced by 6.6%. Moreover, the gas turbine output increases by 6.4%, whereas the steam turbine’s output decreases by 3.4%.

### 4.6. Potential for Efficiency Improvement

**Figure 12:**
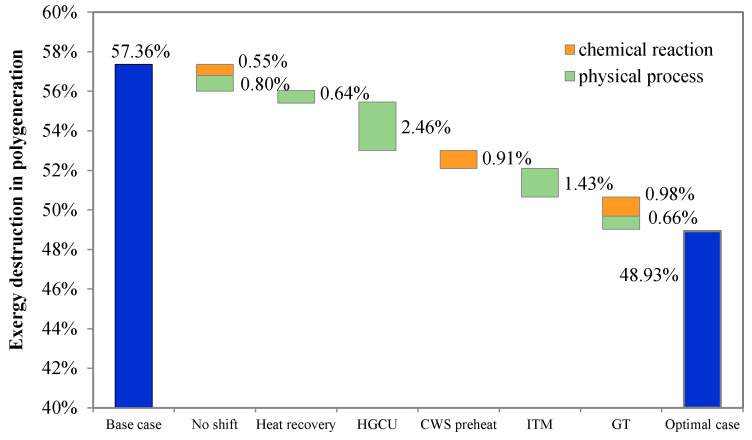
Potential exergy loss reductions for a coal-based polygeneration system.

The impacts of the above technical choices on the efficiency of a polygeneration system are summarized in Figure 12. The overall exergy loss can be reduced significantly through these improvements, from 57.36% to 48.93%. When the water gas shift process is eliminated, the overall exergy loss decreases by 1.35 percentage points compared to the base case. In this reduction, 0.55 percentage points come from chemical reactions, and 0.8 percentage points come from physical processes. Adopting improved gasification technologies contributes significantly to the overall exergy loss reduction in four ways: (1) adding an RSC for heat recovery reduces the system exergy loss by 0.64 percentage points; (2) hot gas cleaning reduces exergy loss the most, by about 2.46 percentage points; (3) integrating coal water slurry pre-heating reduces the exergy loss from chemical processes by 0.91 percentage points; (4) using an ion transport membrane for air separation reduces the overall exergy loss by 1.43 percentage points. Moreover, considering both chemical reactions and physical processes, an advanced G-class gas turbine further reduces the exergy loss by 1.64 percentage points.

## 5. Conclusions

This paper developed an improved structured exergy analysis to reveal the causes of exergy loss in coal-based polygeneration systems. Exergy losses caused by chemical reactions and physical processes were distinguished. An evaluation indicator was proposed to quantify the performance of the physical exergy utilization. Based on this exergy analysis, technologies and systems designs for gasification, methanol synthesis, and gas turbines were investigated for their potential to improve energy utilization. 

In the base case, a series coal-based polygeneration system with water-gas shift and a once-through methanol reactor, the gasification reactions contribute the most to exergy loss, followed by the combustion reactions in the gas turbine and heat transfer for pre-heating reactants in the gasifier. Compared with the base case, the exergy loss can be significantly reduced from 57.36% to 48.93% by adopting advanced technologies and design optimization. Among the four proposed technologies for gasification, hot gas cleaning reduces exergy loss the most (2.46 percentage points), followed by the ion transport membrane technology for air separation (1.47 percentage points), slurry pre-heating (0.91 percentage points), and syngas heat recovery (0.64 percentage points). For the methanol synthesis process, employing a partial shift can reduce the overall exergy loss by about 1.35 percentage points, increasing both overall and physical exergy efficiency. Although unreacted gas circulation works against physical exergy utilization, it benefits overall exergy efficiency due to more methanol production. Finally, employing a G-class gas turbine reduces the exergy losses caused by chemical reactions and physical processes by 0.98 and 0.66 percentage points, respectively. In general, a coal polygeneration system employing all these advanced technologies has an exergy efficiency of 51%, which is 8.4 percentage points higher than that of an unimproved system. 

## Figures and Tables

**Table 1:** Operating parameters and specifications of the model.

Process Name	Simulation Model	Operation Parameters and Specifications
Gasifier	RYield for decomposition; RGibbs for gasification	13,000 C, 6.0 MPa; moisture 14%, VM 36.49%, Ash 12%; C 61.45%, H 3.61%, N 0.71%, O 7.8%, S 0.43%; LHV 23.42 MJ/kg (as received basis)
Water quench	Mixer and Flash2	cold water 1100 C, 6.2 MPa; Flash2 2300 C, 5.9 MPa
Scrubber	RadFrac	pressure of top stage 5.8 MPa
Water-gas shift	RStoic	2500 C, 5.8 MPa
Syngas cooling	Heater	380 C, 5.7 MPa
Acid gas removal (AGR)	RadFrac; MHeatX	380 C, 3.8 MPa; 0.06 MPa
Methanol synthesis	RCSTR; MHeatX; Compr	2500 C, 5.5 MPa; reactant temp. 2350 C
Distillation	RadFrac; MHeatX; Flash2	pre-distillation 0.14 MPa; high pressure distillation 0.56 MPa; normal-pressure distillation 0.12 MPa
Gas turbine	GE 9531 FA	13270C, 15.8 (pressure ratio); IGV −8%
HRSG	Economizer; vaporizer; superheater; pump	5650 C/5650 C/12.5 MPa/2.5 MPa/0.25 MPa
Steam turbine	Turbine	5650 C/5650 C/12.5 MPa/2.5 MPa/0.25 MPa

**Table 2:** Comparison of simulation results of gasifier model with references.

Data Sources	Parameter	CO	H_2_	CO_2_	H_2_O
Monaghan [22] coal 1: Illinois #6	reference	44.88%	38.46%	15.48%	-
	simulation	42.60%	38.00%	15.49%	-
Monaghan [22] coal 2: Pittsburgh	reference	44.27%	39.42%	15.47%	-
	simulation	46.64%	37.92%	12.56%	-
Monaghan [22] coal 3: Limington	reference	44.20%	36.86%	17.95%	-
	simulation	47.42%	36.48%	13.74%	-
Kunze [23]	reference	49.30%	35.80%	12.30%	-
	simulation	49.61%	34.62%	12.50%	-
Wu [24]	reference	32.40%	25.70%	16.10%	25.30%
	simulation	33.87%	27.16%	9.75%	27.94%

**Table 3:** Comparison of simulation results of methanol synthesis model with reference.

No.	Operating Conditions of Zhao [25]				Methanol Equivalent Productivity (mol/kg/h)	
	Temperature (°C)	Pressure (Psig)	Gas Hourly Space Velocity (NL/kg·h)	Slurry Concentration (%)	Experimental Results	Simulation Results
1	250.2	752.4	10,841	36.5	29.9	32.43
2	250.0	753.0	6168	36.5	20.7	24.21
3	250.1	763.6	13,684	37.0	34.1	36.39
4	250.2	752.6	2985	36.5	11.6	14.87
5	235.3	753.0	10,953	35.9	24.1	27.31
6	285.0	753.0	2076	36.6	4.6	12.35
7	249.8	752.7	11,024	35.6	28.6	32.55
8	249.6	753.0	10,327	37.6	26.4	31.58
9	250.2	752.0	11,024	34.8	28.3	26.02
10	249.7	893.5	11,085	35.8	31.1	32.76

**Table 4:** Comparison of exergy losses with heat recovery (unit: % of total exergy input).

Case	Base Case	Case w/RSC	Difference
Heat transfer in quench	6.574	3.387	−3.187
Heat transfer in RSC	0	1.759	+1.759
Heat release in low-T cooling	3.361	1.773	−1.588
Heat transfer in HRSG	1.706	2.549	+0.843
Exhaust and other in HRSG	0.400	0.999	+0.598
Total loss in physical processes	37.264	35.792	−1.472

**Table 5:** Comparison of exergy loss in two once-through series cases (unit: % of total exergy input).

Exergy Loss	Base Case	w/o Shift	Difference
***Chemical reactions***	***21.05***	***20.50***	***−0.55***
Gasification	13.02	13.02	0
Water gas shift	**0.95**	**0**	**−0.95**
Methanol synthesis	**0.56**	**0.68**	**+0.12**
Combustion in gas turbine	6.51	6.79	+0.28
***Physical process***	***36.31***	***35.51***	***−0.80***
Heat transfer in gasifier	6.10	6.10	0
Slag loss, heat release of gasifier	4.30	4.30	0
Air separation process	2.05	2.05	0
Heat transfer in water quenching	6.57	6.57	0
Heat release in low-T cooling	2.81	3.36	+0.55
Loss in acid gas removal	**2.16**	**1.06**	**−1.10**
Heat release of shift reactor	**1.29**	**0**	**−1.29**
Heat exergy release of lpmeoh	0.88	0.85	−0.03
Loss in distillation	0.17	0.11	−0.06
Heat transfer in combustor	3.90	4.29	+0.39
Technical loss of gas turbine	3.35	3.62	+0.27
Heat transfer in HRSG	1.34	1.71	+0.37
Exhaust heat	1.03	1.07	+0.04
Technical loss of steam turbine	0.33	0.40	+0.07

## Data Availability

Not applicable.

## Sample Availability

Samples of the compounds are not available from the authors.

## Nomenclature

AGR	acid gas removal
ASU	air separation unit
CGC	conventional gas cleaning technology
HGC	hot gas cleaning technology
IGCC	integrated gasification combined cycle
ITM	ion transport membrane technology for air separation
*η*	exergy efficiency
*φ*	available exergy utilization rate
*λ*	unreacted gas circulation ratio
*R*	molar gas constant
*E*	exergy of one stream
*E^Q^*	thermal exergy of one heat stream
*E_coal, in_*	exergy of the coal input of gasifier
*E*_O2*, in*_	exergy of the pure oxygen input to one facility
*E_syngas, in_*	exergy of syngas input to one facility
*E_syngas, out_*	exergy of syngas out of one facility
*E_acidgas, out_*	exergy of acid gas out of one facility
*E_slag, out_*	exergy of slag out of gasifier
*E_in_*	exergy of reactants of one chemical reaction
*E_out_*	exergy of products of one chemical reaction
*E_water_*	exergy of water input to water tube of gasifier
*E_steam_*	exergy of steam produced in water tube of gasifier
*E_water,in_*	exergy of water input stream for quenching
*E_water,out_*	exergy of water output stream after quenching
*E^Q^_latent_*	exergy of the latent heat of one heat stream
*E^Q^_sensible_*	exergy of the sensible heat of one heat stream
*E^W^_AGR_*	theoretical minimal separation work
*n_CO_*_2_	mole number of CO_2_ of syngas stream
*n_other_*	mole number of components other than CO_2_ of syngas stream
*Ep*	exergy of electricity
*Ec*	exergy of methanol product
*E_total_*	exergy of overall products of polygeneration system
*I_chem_*	exergy destruction caused by chemical reaction
*I_heating_*	exergy destruction caused by heat transfer processes
*I_Qloss_*	exergy destruction caused by heat loss to the environment
*T*_0_	ambient temperature
*T*	syngas temperature
*h_in_*	enthalpy of the input stream
*h_out_*	enthalpy of the output stream
*S_in_*	entropy of the inputstream
*S_out_*	entropy of the output stream
∆*H*	enthalpy change of one chemical reaction
∆_s*heating*_	entropy production of heating processes
*Q_loss_*	thermal energy of one heat stream
